# High-Density Genetic Map Construction and Identification of QTLs Controlling Leaf Abscission Trait in *Poncirus trifoliata*

**DOI:** 10.3390/ijms22115723

**Published:** 2021-05-27

**Authors:** Yuan-Yuan Xu, Sheng-Rui Liu, Zhi-Meng Gan, Ren-Fang Zeng, Jin-Zhi Zhang, Chun-Gen Hu

**Affiliations:** Laboratory of Horticultural Plant Biology, College of Horticulture and Forestry Science, Huazhong Agricultural University, Wuhan 430070, China; yuanyuanyuan@webmail.hzau.edu.cn (Y.-Y.X.); liushengrui@ahau.edu.cn (S.-R.L.); zhimenggan@webmail.hzau.edu.cn (Z.-M.G.); renfzeng@mail.hzau.edu.cn (R.-F.Z.)

**Keywords:** citrus, SSR, SNP, SLAF-seq, genetic map, leaf abscission, QTL mapping

## Abstract

A high-density genetic linkage map is essential for genetic and genomic studies including QTL mapping, genome assembly, and comparative genomic analysis. Here, we constructed a citrus high-density linkage map using SSR and SNP markers, which are evenly distributed across the citrus genome. The integrated linkage map contains 4163 markers with an average distance of 1.12 cM. The female and male linkage maps contain 1478 and 2976 markers with genetic lengths of 1093.90 cM and 1227.03 cM, respectively. Meanwhile, a genetic map comparison demonstrates that the linear order of common markers is highly conserved between the clementine mandarin and *Poncirus trifoliata*. Based on this high-density integrated citrus genetic map and two years of deciduous phenotypic data, two loci conferring leaf abscission phenotypic variation were detected on scaffold 1 (including 36 genes) and scaffold 8 (including 107 genes) using association analysis. Moreover, the expression patterns of 30 candidate genes were investigated under cold stress conditions because cold temperature is closely linked with the deciduous trait. The developed high-density genetic map will facilitate QTL mapping and genomic studies, and the localization of the leaf abscission deciduous trait will be valuable for understanding the mechanism of this deciduous trait and citrus breeding.

## 1. Introduction

Citrus is one of the most important fruit crops worldwide in terms of fruit production and economical value. In general, citrus includes species from six closely related genera: *Citrus*, *Poncirus*, *Fortunella*, *Eremocitrus*, *Microcitrus*, and *Clymenia* [1]. All of the genera have persistent unifoliolate leaves and are evergreen except for the genus *Poncirus*, which has trifoliate and deciduous leaves. It is reported that the deciduous trifoliate orange (*Poncirus trifoliata*) and evergreen clementine mandarin belong to the same ancestor and originated in subtropical or tropical regions of Asia [2]. During the course of differentiation, trifoliate orange has become deciduous, which may contribute to its tolerance of lower temperatures compared with evergreen citrus [3]. An important feature of trifoliate orange is its cold tolerance [3]. Trifoliate orange is extremely cold-hardy and can survive at −26 °C [4]. 

*Poncirus* is a valuable resource because it possesses genes conferring many agriculturally important and advantageous traits, such as resistance to citrus tristeza virus (CTV), citrus nematode (CN), *Phytophthora* root rot, and tolerance of low temperatures [5]. Moreover, the great advantage of the dominant trifoliate leaf trait of *Poncirus* over the monofoliate of *Citrus* is that it allows zygotic hybrids to be visually distinguished from nucellar seedlings [5]. In citrus, traditional breeding is difficult due to its biological characteristics, such as large plant size, long juvenility, gametophytic cross-incompatibility, heterozygosis, and apoximis [6,7]. In recent years, marker-assisted selection (MAS) has become an efficient strategy to avoid these obstructions. Highly saturated genetic maps are a prerequisite for accurate quantitative trait loci (QTLs) associated with important agronomical traits as well as for providing information for evolution and genome organization studies of citrus species. More importantly, the recent release of the clementine mandarin genome also dramatically enhanced the efficiency of MAS research in citrus [8].

In the past two decades, molecular markers have been developed and applied to construct genetic maps of citrus. However, the traditional molecular marker technologies—such as restriction fragment length polymorphism (RFLP), randomly amplified polymorphic DNA (RAPD), amplified fragment length polymorphism (AFLP), cleaved amplified polymorphic sequences (CAPSs), and simple sequence repeats (SSR)—are highly labor-, time-, and cost-intensive [6]. These constructed citrus genetic linkage maps based on traditional molecular markers have a low density of markers and a greater intermarker distance [1,5,9,10,11]. Recently, large-scale nucleotide polymorphism (SNP) markers utilizing high-throughput sequencing technologies have been developed and used successfully for the construction of high-density genetic maps in citrus species such as the clementine mandarin, sweet orange, and pummelo [6,12]. However, the high-density trifoliate orange genetic map is still absent compared with other citrus species. 

Leaf abscission is a physiological process often associated with stress and senescence [13]. Studies have elucidated that leaf abscission is an important milestone for plant evolution processes that influences the differentiation and geographic distribution of plant species [14,15]. So far, several hypotheses have been proposed for the advantages of having a longer or shorter leaf life span, which are most notably those related to environmental stimuli or developmental timing [16]. For citrus leaf abscission, mature leaves are shed through the activation of the laminar abscission zones located at the interface between the petiole and the leaf blade, and aged leaves by the activation of the branch abscission zone located at the branch to petiole junction [17]. The abscission of citrus leaves is also regulated by developmental, hormonal, and environmental cues [18,19]. A series of stressful environmental conditions such as salinity, subfreezing temperatures, and water stress have been reported to stimulate leaf abscission [17,19,20,21]. Meanwhile, critical functional and global expression studies using microarrays with several platforms have been conducted [17,19]. There is increasing evidence suggesting that there are many members of different gene families related to citrus leaf abscission and involved in hormone metabolic and environmental stimuli processes [17,18,19,22]. However, the genes that play pivotal roles in regulating leaf abscission are still unknown. The recent release of the citrus genome sequence has dramatically enhanced the efficiency of functional and comparative genomics research in citrus [8]. Thus, to gain further insight into evolution and environmental adaptation in citrus, it is important to use the available genome sequence to understand the molecular regulation mechanisms of citrus leaf abscission.

In this study, we generated an F1 segregating population containing 200 progenies derived from the clementine mandarin and trifoliate orange. A high-density integrated genetic linkage map as well as female and male genetic maps were constructed using the SSR and SNP markers, respectively. Two major QTLs were discovered based on the mutmap-like strategy, and a total of 143 candidate genes were obtained based on association analysis. The functional annotation of these candidate genes offer further understanding of their potential roles, and some candidate genes were selected for analyzing their expression patterns under cold stress treatment. In short, QTL mapping for the leaf abscission trait will be of significant use while contributing to the basic understanding of plant biology, and it is also a trait of agricultural importance that has frequently been selected during domestication. Meanwhile, the constructed high-density genetic maps provide valuable information for genome organization, genome comparison, and QTL mapping studies in citrus. 

## 2. Results

### 2.1. Morphological Characterization of Citrus Leaf Abscission and Hormone Quantification

**Figure 1:**
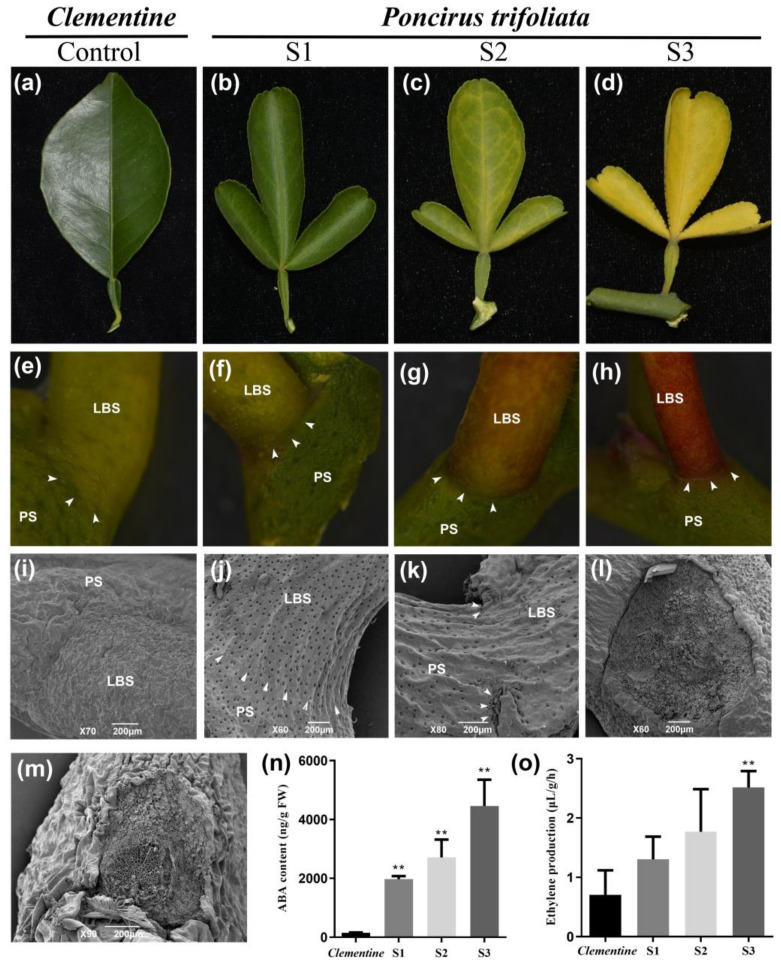
Morphological characterization of citrus leaf abscission and hormone quantification. (**a**) Phenotypic characteristics of clementine mandarin leaves at stage 1 (before abscission). (**b**–**d**) Phenotypic characteristics of trifoliate orange leaves at stage 1 (**b**), stage 2 (**c**), and stage 3 (**d**). Stage 1: before abscission; stage 2: beginning of abscission; stage 3: after abscission. (**e**–**h**) Detailed characteristics of trifoliate orange leaf abscission zone at stage 1 (**f**), stage 2 (**g**), and stage 3 (**h**). (**i**) Cellular morphology of clementine mandarin leaf abscission zone at stage 1. (**j**–**l**) Cellular morphology of trifoliate orange leaf abscission zone at stage 1 (**j**), stage 2 (**k**), and stage 3 (petiole side, **l**), respectively. (**m**) Cellular morphology of clementine mandarin leaf abscission zone (petiole side) after forcible separation. (**n**) Changes in ABA content (ng/g fresh weight) in leaf abscission zone of trifoliate orange. (**o**) Changes in ethylene (μL/g/h) in the leaf abscission zone of trifoliate orange. leaf blade side: LBS; petiole side: PS. The white arrow indicates location of the abscission zone. Asterisks indicate significant differences: ** *p* < 0.01.

To examine the mechanisms of seasonal leaf abscission in citrus, two citrus cultivars were used—the trifoliate orange (deciduous) and clementine mandarin (evergreen). Changes in leaf morphology were investigated at the abscission stage under field conditions (Figure 1). The result showed that the leaves of the trifoliate orange gradually turned yellow and fell off (Figure 1b–d). The leaves of the clementine mandarin were still green throughout the observation period, and no differences were observed (Figure 1a). The leaf abscission process of trifoliate orange was divided into three typical stages based on morphological changes (stage 1: before abscission; stage 2: beginning of abscission; stage 3: after abscission) (Figure 1f–h). The formation of the abscission zone occurred at stage 2 in trifoliate orange (Figure 1g); the leaf blade fell off at the slightest touch at stage 3 (Figure 1h). To examine the changes in the cellular morphology of the abscission zone at all three stages, scanning electron microscopy (SEM) was used. The results revealed that a visible abscission zone was formed between the distal (leaf blade side) and the proximal (petiole side, Figure 1k) after yellowing occurred (Figure 1k). The cells of the proximal fracture planes were showed rounded and elongated and seemed to be loosely attached to one another (Figure 1l). In the clementine mandarin, the fracture plane showed a ragged surface of broken cell walls, indicating that forcible separation results in the breaking of primary walls due to the high cell adhesion strength in the abscission zone (Figure 1m). 

ABA and ethylene are the two important hormones regulating the organ abscission of plants. Thus, the concentrations of endogenous ABA and ethylene in the leaf abscission zone were determined during the leaf abscission process (Figure 1n,o). The results show that these two hormones gradually increase during the abscission process. However, the content of these two hormones is lower in the clementine mandarin (Figure 1n,o). These results indicate that these two hormones may play an important role in the leaf abscission process.

### 2.2. Hybrid Population Construction

**Figure 2:**
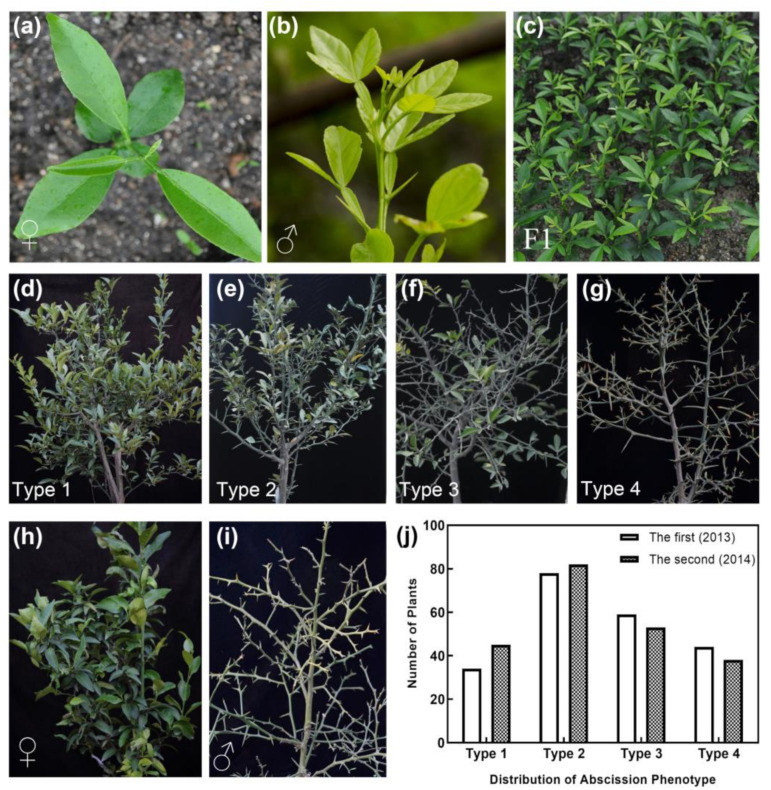
Hybrid population construction. (**a**) Clementine mandarin (female parent) at the seedling stage. (**b**) Trifoliate orange (male parent) at the seedling stage. (**c**) F1 hybrid population at the seedling stage. (**d**) Hybrids with no leaf abscission phenotype (Type 1). (**e**) Hybrids with slight leaf abscission phenotype (Type 2). (**f**) Hybrids with a severe leaf abscission phenotype (Type 3). (**g**) Hybrids with all leaves dropped (Type 4). (**h**) The evergreen phenotype of the female parent (clementine mandarin). (**i**) The deciduous phenotype of the male parent (trifoliate orange). (**j**) The statistics data of the leaf abscission phenotype variation of progeny type 1 to type 4 represent the (**d**) to (**g**) different phenotypes, respectively.

To investigate the leaf abscission genetic characteristics, 316 F1 individuals were obtained after pollination between the clementine mandarin (female, Figure 2a) and trifoliate orange (male, Figure 2b). The deciduous degree of hybrids was evaluated in the field and divided into four types based on the leaf abscission trait of the male parent (Figure 2h) and female parent (Figure 2i). Type 1: Hybrids with no leaf abscission phenotype (Figure 2d) Type 2: Hybrids with a slight leaf abscission phenotype (intermediate traits, Figure 2e). Type 3: Hybrids with a severe leaf abscission phenotype (intermediate traits, Figure 2f). Type 4: Hybrids with all leaves dropped (Figure 2g). The degree of deciduousness for each individual was highly consistent within two years (Figure 2j) and there are four distinct deciduous degree phenotypes suggesting that the deciduous trait is likely to be controlled by quantitative trait loci. The F1 population had wide segregations for the phenotypic traits, which indicated that the population was suitable for the mapping population.

### 2.3. Screening and Verification of SSR and InDel Markers

**Figure 3:**
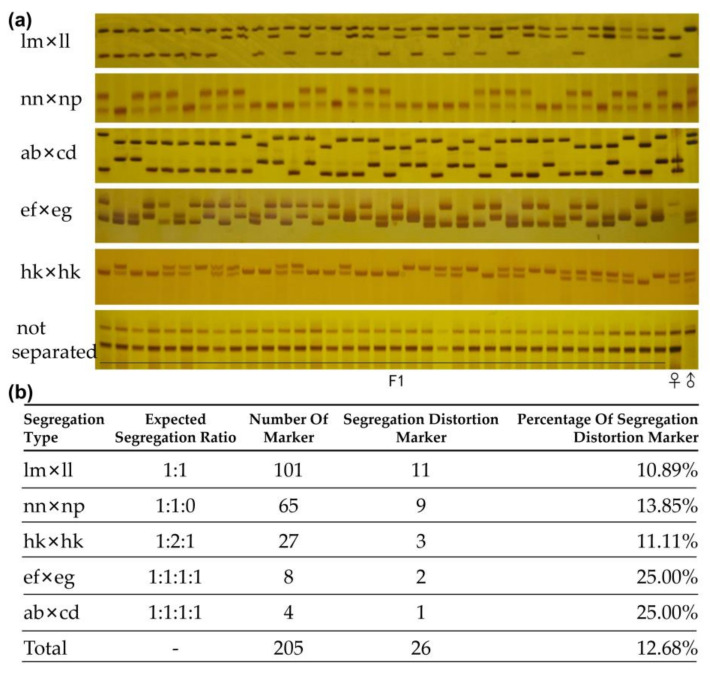
Screening and verification of SSR and InDel markers. (**a**) Five types of segregation and one type of null amplification for 36 hybrid progenies from the F1 hybrid population. (**b**) Summary of the segregation types and distorted segregation markers.

To identify the citrus deciduous trait-related QTLs, a total of 774 pairs of primers of SSR (700) and InDel (74) markers were screened using six randomly selected F1 hybrid individuals and the two parents. Subsequently, five segregation types and one type of null allele were demonstrated with the F1 population based on the pseudo-testcross mapping strategy (Figure 3a). Finally, 205 markers (194 SSRs and 11 InDels) were identified and qualified for genetic map construction after filtering markers of null amplification (obscured amplification, non-amplification, non-specific amplification, non-polymorphic, and non-segregation markers). 

To construct a genetic linkage map using SSR and InDel markers, 205 markers were further screened in 316 F1 hybrid progenies, and finally, the genotypes of all the progeny were identified by these markers (Figure 3b). Among the polymorphic markers, 101 (49.03%) were segregated in female meiosis (lm Screening and Verification of SSR and InDel Markers ll, with the parental genotypes lm × ll, where different letters denote distinct alleles), accounting for the largest proportion. 65 (31.71%) were segregated in male meiosis (nn Screening and Verification of SSR and InDel Markers np), 12 (5.85%) fully informative markers were heterozygous in both parents (four with four distinct alleles, ab×cd type, and eight with three alleles, ef Screening and Verification of SSR and InDel Markers eg), and 27 (13.17%) were partially informative in both parents (hk × hk). A further chi-square test was performed on the markers to identify whether the genotype frequency of the locus matched the Mendelian expected segregation ratio (0.001 < *p* < 0.01), and finally, a total of 26 marker genotypes were identified as partial segregation markers, accounting for 12.68% of the total markers (Figure 3b).

### 2.4. SLAF Sequencing and Genotyping

The F1 population comprised of the two parents and 200 progenies was genotyped using specific-locus amplified fragment sequencing (SLAF-seq) technology [23]. In total, 97.76 Gb of data containing 489.33M pair-end reads was obtained with each read being 125 bp in length (Table 1). Among them, the female and male parental datasets contained approximately 1.62 Gb (comprising 7,664,165 reads with a GC% of 39.00) and approximately 1.53 Gb (comprising 8,095,110 reads with a GC% of 37.98), respectively. From the 200 progenies, a total of 473.58M reads corresponding to approximately 94.61 Gb of data with a GC% of 38.66 were generated. 

**Figure 4:**
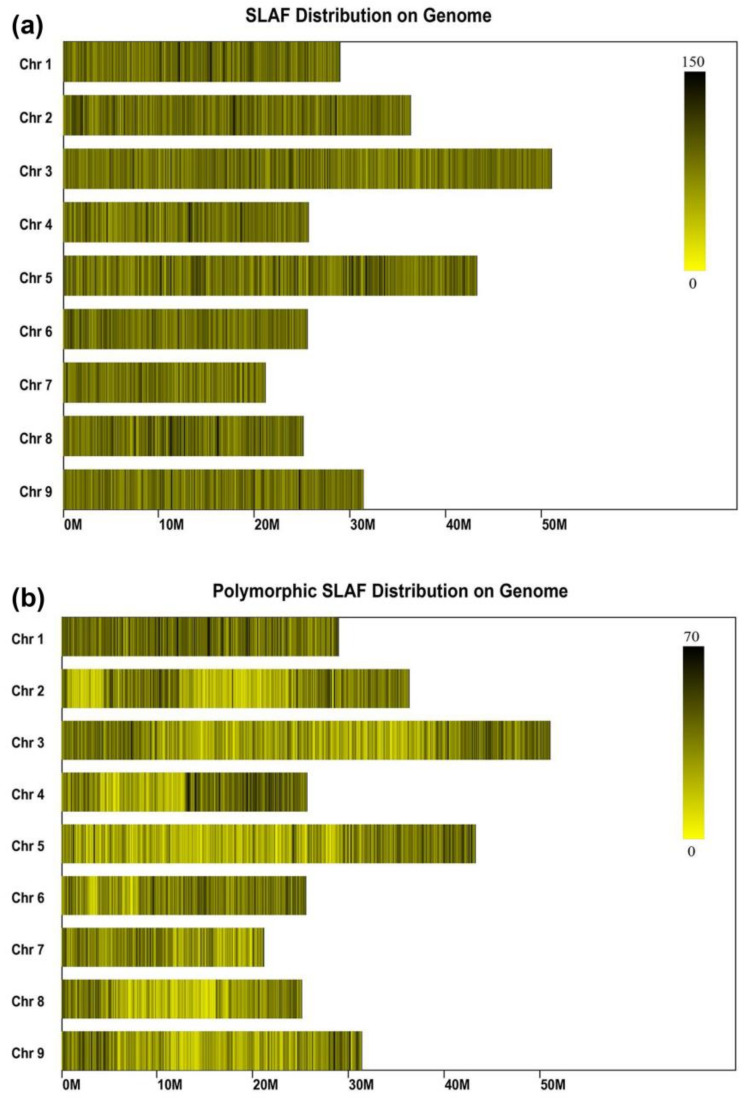
SLAF sequencing and genotyping. (**a**) The distribution of all obtained the SLAF tags on nine citrus chromosomes. (**b**) The distribution of all the polymorphic SLAF tags on nine citrus chromosomes. The x-axis represents the location on chromosomes, the y-axis represents the nine chromosomes, the x-axis represents the length of chromosome. When the color is darker, the SLAF tag number is greater.

The obtained reads were subsequently mapped to the clementine mandarin genome (https://phytozome.jgi.doe.gov/pz/portal.html#!info?alias=Org_Cclementina), and the reads that could be mapped to a single locus were regarded as effective SLAFs. The results showed that all the SLAF tags obtained after comparison with the reference genome covered almost all the regions of the nine chromosomes of citrus, and the overall distribution was relatively uniform (Figure 4a). Although the polymorphic SLAF tag covers almost all regions of the nine chromosomes, its distribution was less uniform (Figure 4b). A total of 257,409 high-quality SLAFs were obtained consisting of polymorphic, non-polymorphic, and repetitive types (Table 1). Among them, 84,525 were polymorphic with a polymorphism rate of 32.84%, and 24,733 SLAFs (divided into eight different separation types) were successfully genotyped in both parents and progeny (Figure S1). The average sequence coverage for each SLAF marker was 37.07-fold for the female parent and 39.81-fold for the male parent. For the F1 population, the average read number for SLAFs was 1,420,829, with an average SLAF number of 146,644, and the average coverage was 9.69-fold. The SLAFs were further screened to discard markers that were inappropriate for genetic map construction (16,238 markers belonging to the aa × bb segregation type were filtered out because they were inappropriate for the cross pollinator population), and 8495 SLAFs could be applied for genetic map construction. To guarantee the quality of the genetic linkage map, stringent parameters were used to screen these SLAFs. Eventually, a total of 4307 SLAFs were obtained for the construction of the genetic linkage map.

### 2.5. High-Density Linkage Map Construction Using SNP, SSR, and InDel Markers

A total of 4307 SNPs, 194 SSRs, and 11 InDels were loaded into Highmap for linkage analysis. After grouping, a total of 4163 markers (3991 SNPs, 163 SSRs, and 9 InDels) were successfully distributed into the consensus nine LGs with 1393 markers located in distinct genetic positions. All the SLAF sequences that possessed the 3991 SNPs were obtained (Table S1), and five segregated genotypes of all of the 4163 markers were demonstrated (Table S2). The ratio of successfully mapped markers of SNPs, SSRs, and InDels was 92.66%, 84.02%, and 81.82%, respectively. Among all the markers successfully genotyped, the five segregation patterns of nn × np, lm × ll, ef × eg, ab × cd, and hk × hk were encountered for 1187, 2685, 51, 240, and 0 (only three hk × hk segregation SSR markers were obtained and unsuccessfully used for linkage analysis), respectively.

The sex-specific genetic maps were constructed using markers that were heterozygous in only the female or male parent. The maternal genetic map constituted 1478 markers (477 unique markers), with a total genetic distance of 1093.90 cM (Table 2, Figure S2). The marker intervals based on the unique marker positions ranged from 1.43 cM (LG3) to 4.47 cM (LG8) with an average marker interval of 2.66 cM. The paternal genetic map consisted of 2976 markers (1017 unique markers), with a total of genetic length of 1227.03 cM, which was much longer than the maternal genetic map (Table 2, Figure S3). The marker intervals of the paternal genetic map ranged from 1.02 cM (LG1) to 3.15 cM (LG8), with an average of 1.38 cM between adjacent loci.

**Figure 5:**
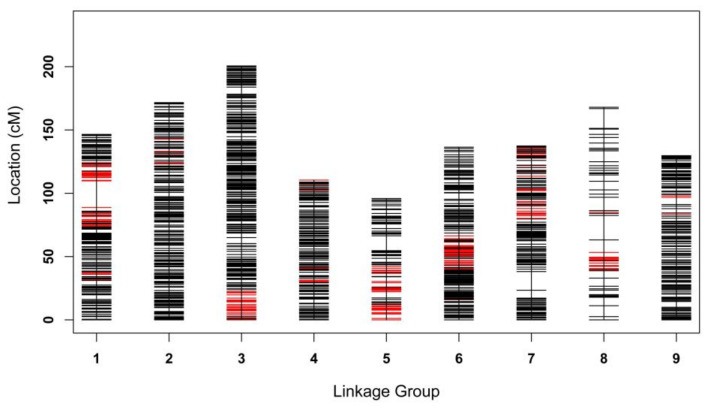
Distribution of all polymorphic SSR and SLAF markers in nine linkage groups. The x-axis represents each LG, the y-axis represents the genetic distance of LGs, the red line represents segregation distortion markers (0.001 < *p* < 0.05).

The integrated map was constructed using markers that were heterozygous in both parents (Figure 5 and Table 3). As a result, a total of 4163 markers were mapped, which comprised 1393 markers with distinct genetic positions. The integrated map spanned 1296.3 cM with an average genetic distance of 1.12 cM between adjacent loci, ranging from 0.75 cM (LG6) to 3.17 cM (LG8). The max gap ranged significantly, from 3.26 cM (LG4) to 20.99 cM (LG1), and the gap smaller than 5 cM accounted for more than 98% of each LG. LG3 contained the highest number of markers, while LG8 consisted of the fewest markers, with the largest average interval between loci of 3.17 cM. To ensure the high quality of the map and highly accurate QTL mapping, skewed markers with non-Mendelian markers (*p* < 0.001) were discarded. The ratio of skewed marker (0.001 < *p* < 0.05) on each LG was evaluated. As shown in Table 3 and Figure 5, the ratio of distorted markers ranged from 2.2% (LG2) to 44.9% (LG5), with an average of 16.1%, suggesting that segregation distortion markers are unevenly distributed on LGs. It is worth mentioning that most distorted markers formed clusters on some LGs, such as LG1, LG3, LG5, LG6, and LG8, suggesting that these are likely to be segregation distortion regions.

### 2.6. Comparative Analysis of the High-Density Linkage Map

**Figure 6:**
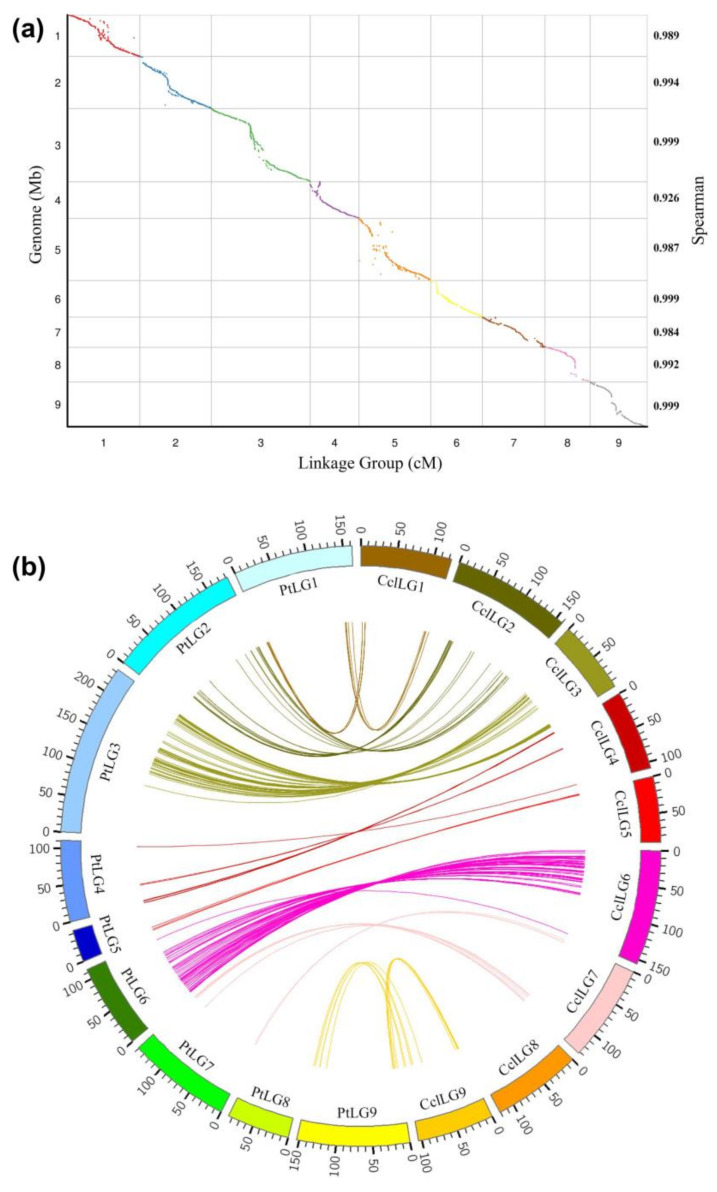
High-density genetic linkage map construction of citrus. (**a**) Collinearity analysis of all citrus linkage groups with its genome sequence. The x-axis scales the genetic distance of citrus LGs accordingly: the left y-axis represents the position of citrus pseudo-chromosomes and the right y-axis represents the Spearman correlation coefficient. SLAF markers in these LGs are plotted as dots. (**b**) Conservation of the synteny and linear order of markers between the female and male parent. Each colored arc represents an orthologous match between two species. Ccl: Clementine mandarin, Pt: *Poncirus trifoliata*.

Collinearity is an important indicator of the quality of a linkage map and is based on the marker order in linkage maps compared to the locations on the citrus reference genome. Therefore, to evaluate the quality of the genetic map, SLAF markers were firstly mapped to the clementine mandarin genome. As a consequence, a collinearity analysis was performed between the physical distances and genetic distances of all markers in the nine LGs (Figure 6a). All of the nine Spearman correlation coefficients were greater than 0.97 based on the collinearity analysis. The consecutive curves found for the nine LGs indicate that the citrus genome was sufficiently covered with SLAF markers, and that the SLAF markers were distributed accurately over each LG. Most parts of these curves represent a falling trend, supporting the notion that their genetic and physical positions follow an identical order. Remarkably, our observations showed that several potential recombination hotspots exist based on collinearity analysis, such as at LG2, LG3, and LG5.

Synteny was considered as the collocation of markers in the same chromosome and completely conserved between all of the parental genetic maps. Moreover, genetic map comparisons could not be conducted based on dominant and mainly cross-specific markers, such as RAPD, AFLP and ISSR. However, multi-allelic co-dominant markers of SSRs and biallelic co-dominant SNPs are powerful in genetic map comparisons. Consequently, a total of 294 common markers can be used for genetic map comparison (with the majority of common markers located on LG3 and LG6), and the linear order of these common markers was highly conserved between the clementine mandarin and trifoliate orange (Figure 6b), with only a few cases of inverted order in small intervals. However, the genetic distance between markers appeared to be unequal between the two parents. Distinct genetic distance between the parents can be caused by several factors, such as their distinct genome sizes, the bias of the different number of loci between the parents, and small genotyping errors concerning the markers located in these regions.

### 2.7. QTL Mapping of Leaf Abscission Trait in Citrus

In order to investigate the leaf abscission genetic characteristics, we first analyzed the QTLs controlling leaf abscission using the MapQTL software 5.0 based on the constructed linkage map and phenotypic separation among progenies. The threshold of significance (*p* = 0.05) for each marker after 1000 permutations was set to 3.0. As a result, although some significant peaks were found in the nine linkage groups, most of them were below the significance threshold. Therefore, these peaks were unable to offer reliable QTLs.

**Figure 7:**
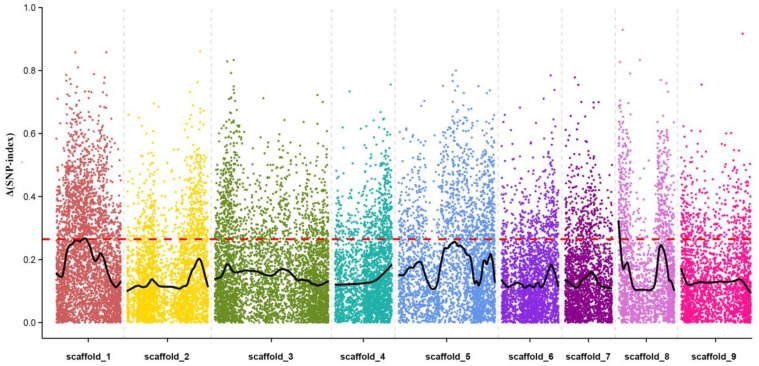
QTL mapping of the leaf abscission trait using the mutmap strategy. The x-axis represents each scaffold of citrus and each colored dot represents an SNP marker. The y-axis represents a Δ(SNP-index) value corresponding to the association degree, the threshold of Δ(SNP-index) value is marked by a red dotted line, and values higher than it were regarded as traits associated with candidate regions.

To conduct the fine mapping of the loci controlling the leaf abscission trait, a mutmap-like strategy was applied to identify the polymorphism of the SLAF markers. The mutmap strategy was developed based on bulked segregation analysis (BSA) by calculating a parameter (SNP-index) to evaluate the contribution of an allele to the mutant phenotype [24]. Here, we considered the evergreen and deciduous traits to be the wild trait (Pool1) and mutant trait (Pool2), respectively. Combining the information from the two graphs for Pool1 and Pool2, we made a graph of Δ(SNP-index) for each SLAF marker on the genome. Ultimately, the chance that Δ(SNP-index) becomes higher than 0.26 was observed for the chromosomal region, and two trait-related candidate regions were obtained on scaffold 1 and scaffold 8 (Figure 7). The obtained QTL regions of the leaf abscission trait for LG1 and LG8 corresponded to the genome interval of 12,177,754 to 13,484,746 bp and 19,579 to 689,575 bp, and contained 36 and 107 genes, respectively (Table S3).

### 2.8. Analysis and Annotation of the Candidate Genes

A total of 143 candidate genes were identified in two candidate regions (Table S3). Among these candidate genes, some genes may be involved in leaf abscission based on the previous reports concerning other plants. For example, several genes encoding endochitinase and expansin associated with cell wall biosynthesis, loosening, and degradation were identified. The expansin activity and its gene expression were significantly induced during ethylene-promoted leaflet abscission in Sambucus nigra [25]. Some candidate genes associated with hormone biosynthesis and signal transduction such as auxin and cytokinin were found in this study. They may also be involved in the regulation of the citrus leaf abscission process based on previous reports [26,27,28]. In addition, eight genes encoding protein kinase were found in two candidate regions. Previous studies indicated that protein kinases were associated with abscission and development [29,30,31]. These results indicate that these candidate genes may be involved in abscission, dehiscence, and other cell separation processes.

**Figure 8:**
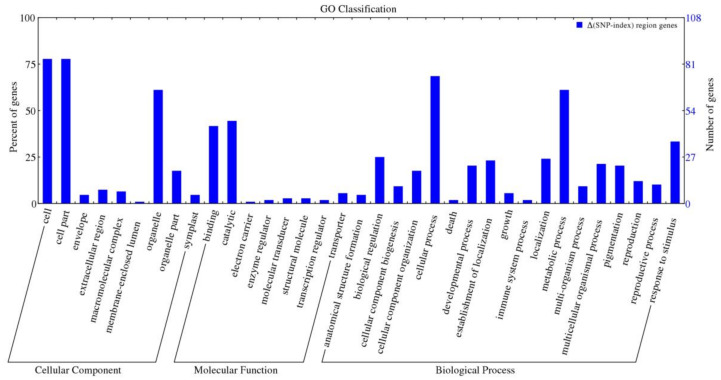
Functional annotation of candidate genes within leaf abscission trait association regions. The x-axis represents the GO classification of three parts, which include the cellular component, molecular function, and biological process, respectively. The y-axes on the left and right represent the percentage and number of genes, respectively.

To further understand the potential functions of these candidate genes, GO annotation was performed using Blast2GO. Based on GO annotation, 141 genes were assigned GO numbers (Figure 8). Cell, cell part, and organelle were the most dominant groups out of these sequences that were annotated to the cellular component category. These were followed by organelle part, extracellular region, macromolecular complex, symplast, envelope, and membrane-enclosed lumen (Figure 8). Catalytic and binding were the most dominant groups that were annotated to the molecular function. However, other groups accounted for a small proportion, such as transporter, transcription regulator, and molecular transducer. With regard to the biological process, cellular process and metabolic process accounted for the two highest groups, which were followed by response to stimulus. In addition to these, biological process, developmental process, multicellular organismal process, and localization also accounted for a high proportion. Some other groups probably offered extremely important information even though they accounted for only a small proportion, such as death, growth, and immune system processes. These annotations reveal that these candidate genes participate in various biological processes and have diverse molecular functions.

### 2.9. Analysis and Annotation of the Candidate Genes

**Figure 9:**
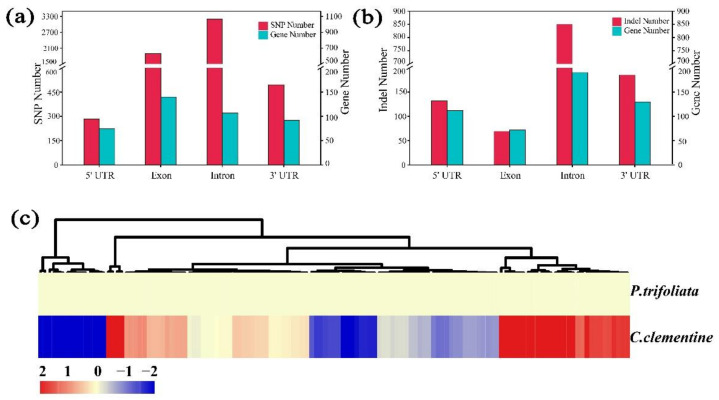
Genetic variation and expression analysis of the candidate genes. (**a**) Numbers of SNP in the candidate genes. (**b**) Numbers of InDel in the candidate genes. (**c**) Heat map of the candidate genes using data from a previous study [33]. Genes highly or weakly expressed in the tissues of mature leaf are colored red and blue, respectively. The heat map was generated using the Cluster 3.0 software. Note: the leaves of the parents were collected at the same developmental stage. Mature leaves represent fully developed leaves rather than senescent and yellowed leaves.

The position of genetic variation within a genome can affect both gene expression and its function. Genetic variants present within coding regions are particularly important as they might alter protein function [32]. Therefore, the discovery of genetic variants related to the functional changes of genes is important for investigating the reasons for phenotypic differences. Based on the whole-genome deep re-sequencing data (40-fold) of parents (SRX1605910) and the citrus reference genome [8], we found a large number of SNPs and InDels in 143 candidate genes (including 5′-UTR, 3′-UTR, intron, and exon regions) after alignments with the clementine mandarin reference genome (Tables S4–S6). A total of 5864 SNPs (283 in 5’ UTR of 75 genes, 3193 in intron of 107 genes, 1893 in exon of 140 genes, and 495 in 3’ UTR of 92 genes: Figure 9a) and 1237 InDels (132 in 5’ UTR of 56 genes, 69 in exon of 36 genes, 851 in intron of 95 genes, and 185 in 3’ UTR of 65 genes: Figure 9b) were identified between parents distributed in 138 and 125 genes, respectively. It is noteworthy that there are five genes (Ciclev10010493m, Ciclev10010759m, Ciclev10010272m, Ciclev10010179m, and Ciclev10010360m) without any genetic variation in their genomic sequences.

To verify whether these candidate genes were differentially expressed between the female parent and male parent, the expression patterns of the 143 candidate genes were also analyzed in the mature leaves of parents using previously reported RNA-seq data (SRP155584) [33]. A total of 131 genes were detected in parents. Compared with *Poncirus trifoliata* (male), 68 upregulated genes and 63 downregulated genes clementine mandarin (female) (Figure 9c). There were 20 genes that were differently expressed, with a probability ≥0.8 and the absolute value of |log_2_^ratio^| ≥ 1 used as the threshold, suggesting that some of these genes may be associated with the leaf abscission trait (Table S8). Among these differentially expressed genes, some genes associated with cell wall biosynthesis, loosening, and degradation were identified, with most of the genes exhibiting significant changes in parents. For example, two genes encoding chitinase and endochitinase protein (Ciclev10028959m and Ciclev10028831m) were found, respectively. One gene encoding expansin protein (Ciclev10029088m) was found to be downexpressed in females, suggesting that it may play an important role in promoting wall dissolution during the leaf abscission process. 

### 2.10. Expression Patterns of Candidate Genes under Cold Stress Treatment

**Figure 10:**
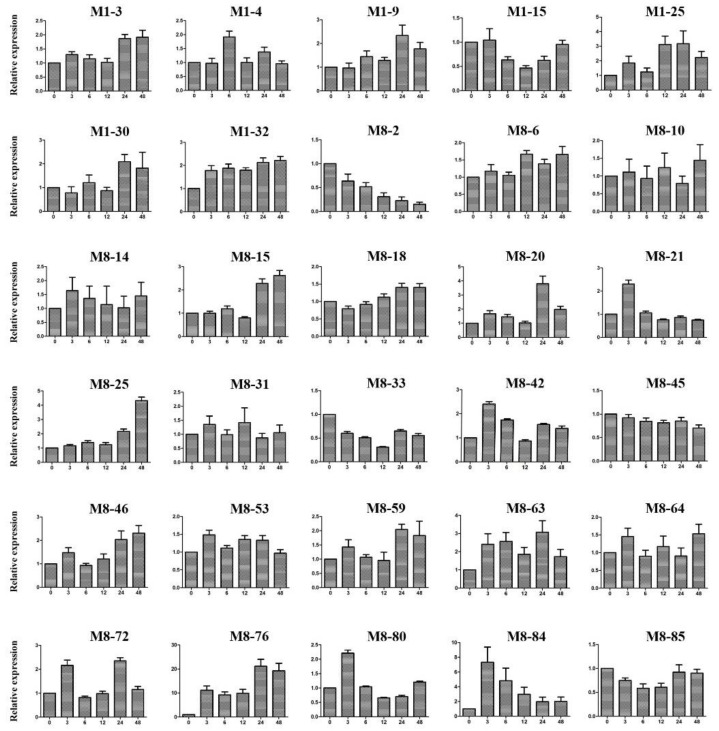
The expression patterns of 30 candidate genes under cold stress conditions. M1-3 (Ciclev10008657m): BREVIS RADIX-like 4; M1-4 (Ciclev10010581m): elongation factor Ts family protein; M1-9 (Ciclev10007290m): outer arm dynein light chain 1 protein; M1-15 (Ciclev10007673m): receptor-like kinase 1; M1-25 (Ciclev10008981m): galactose mutarotase-like superfamily protein; M1-30 (Ciclev10008350m): zinc finger (Ran-binding) family protein; M1-32 (Ciclev10008944m):glutathione S-transferase family protein; M8-2 (Ciclev10027745m): phototropin 2; M8-6 (Ciclev10028759m): pentatricopeptide repeat (PPR) superfamily protein; M8-10 (Ciclev10030351m): ARID/BRIGHT DNA-binding domain-containing protein; M8-14 (Ciclev10028616m): xylem cysteine peptidase 1; M8-15 (Ciclev10028022m): CCAAT-binding factor; M8-18 (Ciclev10028700m): homoserine kinase; M8-20 (Ciclev10028883m): Phosphate transporter 3; M8-21 (Ciclev10028110m): Calmodulin-domain protein kinase 5; M8-25 (Ciclev10028739m): xylem cysteine peptidase 1; M8-31 (Ciclev10029135m): expansin A20; M8-33 (Ciclev10028098m): glutamate tRNA synthetase; M8-42 (Ciclev10028539m): protein kinase superfamily protein; M8-45 (Ciclev10030120m): patched family protein; M8-46 (Ciclev10027787m): pentatricopeptide repeat (PPR) superfamily protein; M8-53 (Ciclev10028324m): protein of unknown function; M8-59 (Ciclev10028533m): lysm domain GPI-anchored protein 2 precursor; M8-63 (Ciclev10027792m): protein of unknown function; M8-64 (Ciclev10028235m): BR-signaling kinase 1; M8-72 (Ciclev10028831m): basic chitinase; M8-76 (Ciclev10029088m): expansin-like A1; M8-80 (Ciclev10028096m): rho guanyl-nucleotide exchange factor 1; M8-84 (Ciclev10028140m): SKU5 similar 5; M8-85 (Ciclev10028628m): chloroplast signal recognition particle component. The number on the x-axis represents the time point after the cold treatment. The y-axis represents the mRNA relative expression level of the gene.

Generally, the leaf abscission phenotype of deciduous plant species has a close relationship with external environmental conditions, such as temperature, light, and water [14,18,34,35]. Among them, low temperature is one of most important factors. Hence, some potential candidate genes were selected for validation based on three principals: the results of functional annotation, genes with difference in sequence between the clementine mandarin and trifoliate orange, and a previous study of transcriptome sequencing for trifoliate orange under cold treatment [36]. These genes may be involved in protein metabolism, hormone biosynthesis and signal transduction, transport and protein phosphorylation, according to previous studies and homology prediction, and they are probably associated with the leaf abscission trait. The expression patterns of 30 selected candidate genes were graphically represented (Figure 10). Remarkably, the expression level of 15 genes was upregulated significantly after cold stress, but they had distinct time points in response to low temperatures. Only three candidate genes were downregulated greatly, and the expression levels of the remaining the 12 genes showed no significant differences after the cold treatment. These genes with a significant change in expression level probably play significant roles in plant development and response to low temperatures, providing us with valuable information for their functional characterization. 

## 3. Discussion

SLAF-seq is considered to be an effective and high-resolution strategy for large-scale SNP discovery and genotyping [23,34]. The SLAF-seq strategy has been applied successfully in various plant species, such as sesame [35], soybean [37], cucumber [38,39], and *Prunus mume* [11]. Most of the earlier citrus genetic maps were constructed based on intergeneric hybrids [1,5]. However, all of these studies suffered from relatively small mapping populations (less than 150 individuals), and many of them even suffered from the dominant markers (RAPD, AFLP, and IRAP) [10,40]. The current integrated genetic map, established from clementine mandarin female and trifoliate orange male segregation, includes 4163 co-dominant markers spread among nine linkage groups. The integrity and accuracy of the developed SLAF markers are high, and the quality and quantity of the markers met the requirements for high-density genetic map construction. The marker density in the current parent maps varies along their genome, and some high-density areas can be observed across the LGs. It is suggested that some high marker density regions are associated with centromeric locations with large physical distances, probably corresponding to low genetic distances [12,41]. Another possibility is that some regions with a high marker density correspond to portions of the genome in interspecific heterozygosity [12]. However, there are some blank or low marker density areas, which probably due to the uneven distribution of the developed markers or reveal highly homozygous regions of the parent genomes. 

To ensure the quality of the genetic map, we discarded those markers of non-Mendalian inheritance (*p* < 0.001). Although markers of non-Mendelian inheritance were excluded, all LGs still observed distortions from the expected Mendelian allelic segregations in this study. The percentage of distorted segregation markers (0.001 < *p* < 0.5) ranged greatly, from 2.2% (LG2) to 46% (LG5), with an average of 16.1%. We found that skewed markers accounted for more than 30% of all markers used for the construction of genetic maps. Marker distortion may be caused by preferential selection or gametic/zygotic selection, which always results in a higher variance [42,43]. In citrus, a study proved that most segregation distortion affects the allele frequencies, representing differences in the pollen fertilization success due to the presence of gametal factors affecting the functionality of gametes and pollen–pistil interactions [9]. It has been reported that skewed markers can be beneficial for QTL mapping because some regions containing skewed markers may be recombination hotspots for specific traits [44,45]. To increase the quantity of markers and the genomic coverage of the genetic map, skewed markers (0.001 < *p* < 0.5) were not excluded in our investigation. The percentage of distorted markers in our study is higher than in previous studies [1,6,13,46], which is likely caused by the myriad of factors involved and needs further investigation. 

To investigate the leaf abscission genetic characteristics, the QTLs controlling leaf abscission were first analyzed with the MapQTL software based on the constructed linkage map and phenotypic separation among progenies. Unfortunately, although some peaks were identified along with nine LGs using QTL mapping, most of them were lower than the threshold value of LOD as 3 and insufficient to offer reliable QTLs. Subsequently, we identified QTLs using the mutmap strategy, which has been proven to be highly efficient [11,24]. Combining a high-density genetic map with two years of deciduous phenotypic data, two QTLs conferring the leaf abscission trait were located by bulked segregation analysis. There are two possible explanations for why the QTLs controlling leaf abscission were not identified by MapQTL analysis. First, leaf abscission is not only regulated by the internal genetic mechanisms of plant development but also by environmental factors, such as drought, pests, temperature, nutrition, and diseases [14,18,20,47], so the phenotype of some individuals may be misjudged during the observation period. Second, only 200 individuals were used to construct a genetic linkage map in this study, so a few individuals with inaccurate phenotypes may seriously interfere with the LOD value. For the mutmap strategy, a total of 30 samples were collected and mixed into a pool of extreme traits, and a few inaccurate phenotypes of individuals were filtered as background noise. Therefore, the mutmap strategy appears to be more effective than QTL mapping analysis in this study.

According to the result of the deciduous degree of progenies, the ratio of evergreen individuals: partial abscission individuals: completely deciduous individuals was approximately 1:2:1. Furthermore, the hybrids of pummelo and trifoliate orange are evergreen [4], which indicates that the evergreen trait is dominant relative to the deciduous trait. Hence, we speculate that at least two major genes significantly control leaf abscission in citrus. Combining the above speculation, these two QTLs may be related to the leaf abscission trait by the mutmap strategy. In a previous study, a genetic analysis of the trifoliate orange deciduous trait was also performed based on QTL mapping [48]. Although a position from trifoliate orange, LG7, was identified that was probably involved in its deciduous trait, the population size and marker number are extremely limited; thereby, we are possibly unable to ensure the accuracy of the identified QTLs [48]. To identify potential candidate genes in this study, we compared the detected QTLs with the clementine mandarin reference genome and extracted some candidate genes in the two regions. There is increasing evidence suggesting that the abscission of leaves is regulated by developmental, hormonal, and environmental cues [14,18,20]. However, the key regulators or genes associated with leaf abscission are still unknown. In addition, the molecular mechanisms of deciduous trifoliate orange are likely distinct from the evergreen citrus leaf abscission under stress conditions. Therefore, it was difficult to determine the leaf abscission controlling the gene directly.

Plant organ abscission involves the co-ordinated breakdown of the cell walls of cells in the abscission zone, and a number of wall-degrading activities have been implicated in this process [25]. It is well established that pivotal processes associated with stress-induced leaf abscission include the degradation and biosynthesis of cell walls in citrus [17,19]. Interestingly, several expansin family genes (Ciclev10029135m, Ciclev10029088m, and Ciclev10029905m) were found in this identified region, and the expression level of (Ciclev10029088m) was upregulated significantly in evergreen females. In citrus, previous study indicated that phosphorylation-related protein kinases were preferentially expressed at the leaf abscission zone after ethylene treatment [18]. Hence, eight protein kinases were identified in our study that may directly or indirectly regulate citrus leaf abscission. Many hormones regulate the process of leaf abscission, such as auxin, ABA, and ethylene [13,27]. As previously reported [13], auxin seems to play a relatively important role in the leaf abscission process, because a total of 10 genes of auxin-induced proteins are located in the association region. Overall, the acquirement of candidate genes will greatly facilitate the identification of leaf abscission-related genes and their functional characterization.

## 4. Materials and Methods

### 4.1. Plant Material

An F1 population of 316 progenies was generated from a cross between the clementine mandarin (female) and trifoliate orange (male). Plants were grown in the field under a natural environment in the farm of Huazhong Agricultural University, Hubei province, China. Leaf samples were collected from each individual F1 plant. Young leaf samples were harvested from each individual F1 plant, immediately frozen in liquid, and kept at −80 °C. A total of 200 randomly selected progenies and the two parents were collected. Genomic DNA was extracted according to a the modified CTAB method [46]. 

Trifoliate orange plants were grown in the greenhouse (16 h light/8 h dark, 25 ± 2 °C) in 20 cm pots containing a commercial potting mix and perlite (3:1, *v/v*). The three months seedlings were selected for 4 °C cold treatment. Leaves were collected at 0, 3, 6, 12, 24, and 48 h after cold treatment. For all the stages, three leaves of each plant from three seedlings were selected. All the samples were immediately frozen in liquid and kept at −80 °C being collected.

### 4.2. Scanning Electron Microscopy (SEM)

The abscission zone of leaves was observed using SEM. To examine the longitudinal sections of the abscission zone, 1 cm portions of tissue were manually dissected with a razor blade. In the clementine mandarin, the peduncle was forcibly separated from the stem. The observation of the abscission zone was carried out as previously described [18]. At least three samples containing the abscission zone were observed.

### 4.3. Quantification of ABA and Ethylene

Leaves of the abscission zone at different developmental stages were used for the determination of ABA and ethylene. The quantification of ABA was determined based on previous reports [49]. The icon isotopes of ABA internal standard (d5-ABA) were used. An Agilent 1100 HPLC (Agilent Technologies, Palo Alto, CA, USA) and API3000 MS-MRM (Applied Biosystems, Foster, CA, USA) were used for the analysis. Each sample was assayed using four replicates. The ethylene production of citrus and tobacco tissues was determined with a gas chromatograph (Hitatchi, Tokyo, Japan) using previously reported methods [50].

### 4.4. SSR and InDel Genotyping 

The sequence information of SSR and InDel primers was obtained from several published papers [13,24,33,43,51]. The SSR markers were screened using six progenies and the two parents, and qualified markers were used to detect the population for constructing citrus genetic maps. For the reaction systems and cycling profile of the PCR amplification, please refer to the previous study [47]. The products of PCR amplification were screened using 6% polyacrylamide gel electrophoresis (PAGE). Band scoring was performed using pBR322 DNA/MapI as a control. The marker code of the polymorphic SSR was analyzed according to the CP (cross-pollinator) population type, which was composed of five segregation types (lm × ll, nn × np, ab × cd, ef × eg, and hk × hk). 

### 4.5. SLAF Library Construction and High-Throughput Sequencing

SLAF library construction and sequencing were performed as described previously [23]. Briefly, a SLAF pre-design in silico simulation experiment was conducted with the genome of clementine mandarin as the reference to establish an optimum enzyme digestion scheme. Then, the SLAF library was constructed according to the pre-designed scheme. Genomic DNA was digested to completion with *Hae* III and *Rsa* I (New England Biolabs, Ipswich, United Kingdom). A single-nucleotide A overhang was added to the digested fragments with Klenow Fragment (3′→ 5′ exo–) (NEB) and dATP at 37 °C; then, the duplex tag-labeled sequencing adapters (PAGE purified, Life Technologies, Carlsbad, CA, USA) were ligated to the A-tailed DNA with T4 DNA ligase. The PCR reaction was performed using diluted restriction–ligation samples: dNTP; Q5^®^ High-Fidelity DNA Polymerase; and the PCR primers: AATGATACGGCGACCACCGA and CAAGCAGAAGACGGCATACG (PAGE purified, Life Technologies). The PCR products were subsequently purified using Agencourt AMPure XP beads (Beckman Coulter, High Wycombe, UK) and pooled. The pooled sample was separated by electrophoresis in 2% agarose gel. Fragments 264~364 bp (with indexes and adaptors) in size were excised and purified using a QIAquick Gel Extraction Kit (QIAGEN, Hilden, Germany). The gel-purified SLAFs were sequenced on the Illumina HiSeq 2500 system (Illumina, Inc; San Diego, CA, USA) at Biomarker Technologies Corporation in Beijing, China (http://www.biomarker.com.cn).

### 4.6. SNP Identification and Genotyping 

The SLAF marker grouping and genotyping were performed according to a previous study [23]. All the SLAF pair-end reads were grouped based on sequence similarity as detected by BLAT (tile size = 10, step size = 5) [52]. Sequences with 90% similarity were clustered in one SLAF locus [35,37]. Alleles were specified to each SLAF according to the minor allele frequency (MAF) evaluation. As citrus is a diploid species, there are at most four kinds of SLAF tags in one locus. Therefore, clusters with over four tags were regarded as repetitive SLAFs and were filtered out with low-depth SLAFs. Only SLAFs with two to four tags were considered polymorphic SLAFs and regarded as potential markers. These potential markers were analyzed as described SSR genotyping. The average sequence depths of the SLAF markers were higher than 38-fold in parents, and higher than 9-fold in progeny. The high-quality SLAF markers for the genetic map construction were discarded by the following criteria: an average depth of sequence less than 10-fold in the parent; an SNP number of more than 8 in each SLAF tag, markers with >30% missing data, and the markers of non-Mendelian inheritance (*p* < 0.001) based on χ^2^ test. 

### 4.7. High-Density Genetic Map Construction

The loci of markers were partitioned primarily into linkage groups based on their locations on the clementine mandarin genome. The modified logarithm of odds (MLOD) score between markers was calculated to confirm the robustness of the markers for each LG, and MLOD scores <5 were filtered prior to ordering. To construct a high-quality genetic map, we used the HighMap strategy to order the SLAF and SSR markers and correct the genotyping within LGs [53]. Firstly, the LOD scores and recombinant frequencies were calculated using the two-point test to infer linkage phases. Subsequently, gibbs sampling, spatial sampling, and simulated annealing algorithms (GSS) were used to perform an iterative process of marker ordering [12,54]. SMOOTH was conducted to correct errors according to the parental contribution of genotypes [55], and the k-nearest neighbor algorithm was used to impute missing genotypes [56]. Then, the skewed markers were added into maps using the multipoint method of maximum likelihood [44]. The sex-specific maps were established using markers that were heterozygous in the female or male parent. The consensus map was constructed by integrating the parental maps through the anchor markers that were heterozygous in both parents [51]. Map distances were calculated using the Kosambi mapping function [57]. 

### 4.8. QTL Mapping for Leaf Abscission Trait in Citrus

Combined with the parental integrated genetic map and phenotypic data, a comprehensive approach for genetic linkage analysis, interval mapping, and cofactor selection and a genome-wide permutation test to detect and map significant QTLs was performed with the MapQTL 5.0 package [58]. Subsequently, the mutmap-like strategy was also used for leaf abscission trait association analysis [24]. High-quality SLAF markers derived from each individual were first divided into two pools (30 individuals for leaf non-abscission and leaf abscission, respectively) according to the phenotypic segregation of the F1 population. The difference in SLAF allele frequencies between two pools was calculated to examine the relative genotype contribution from each parent, further be referred to as the SLAF-index. The following are the calculation formula:

$$\mathrm{SNP}-\mathrm{index}\left(\mathrm{aa}\right)=\frac{aa1}{\left(aa1+aa2\right)}$$

$$\mathrm{SNP}-\mathrm{index}\left(\mathrm{ab}\right)=\frac{ab1}{\left(ab1+ab2\right)}$$

$$∆\left(\mathrm{SNP}-\mathrm{index}\right)=\left[\mathrm{SNP}-\mathrm{index}\left(\mathrm{aa}\right)\right]-\left[\mathrm{SNP}-\mathrm{index}\left(\mathrm{ab}\right)\right]$$

There are two genotypes for each marker: aa1 represents the depth of the aa sample in the first genotype sequence, aa2 represents the depth of the aa sample in the second genotype sequence, ab1 represents the depth of the ab sample in the first genotype sequence, and ab2 represents the depth of the ab sample in the second genotype sequence. Finally, a combined threshold value was calculated for the association analysis.

### 4.9. Functional Annotation of Candidate Genes

To assign the putative functions of the candidate genes, the Blast2GO program was run locally to BLAST against a reference database that stores UniProt entries and their associated Gene Ontology (GO), Enzyme Commission (EC), and Kyoto Encyclopedia of Genes and Genomes (KEGG) annotation [59]. The GO categorization results were expressed as three independent hierarchies for biological process, cellular component, and molecular function.

### 4.10. Real-Time RT-PCR Verification

Total RNAs were isolated with the Oligotex mRNA mini kit (Qiagen, Dusseldorf, Germany) according to the manufacturer’s instructions. The RNA preparation was treated with Dnase I and the first-strand synthesis of cDNA was performed by RT Primer Mix and Primescript RT Enzyme Mix I. There were primer lengths of 20–22 bp and amplicon lengths of 91–242 bp (Table S1). Real-time RT-PCR was conducted using the LightCyclerW480 Detection System (Roche, Basel, Switzerland). To normalize the variance among samples, the expression level of the citrus *β-actin* was used as an internal reference [54]. One-way ANOVA was performed with SPSS to obtain the *p* values. 

## 5. Conclusions

Here, a high-density genetic map of citrus was constructed using SSR and SNP markers, containing 4163 markers with an average distance of 1.12 cM between loci and providing invaluable resources for further QTL mapping and genome studies in citrus. Two trait-related candidate regions were obtained on scaffold 1 and scaffold 8. The two candidate regions had a size of 1.98 Mb and contained 36 and 107 genes, respectively. The functional annotation of these candidate genes suggested that they may be involved in numerous metabolic and physiological processes of leaf abscission. A large number of genetic variations were identified in these candidate genes based on the genome re-sequencing of parents. Moreover, the expression patterns of 30 candidate genes were investigated under cold stress conditions because cold temperatures are closely linked with the deciduous trait. Overall, the successful QTL mapping of the leaf abscission trait is pivotal for map-based gene cloning and understanding the mechanism of the deciduous trait. The acquirement of a candidate gene will greatly facilitate the identification of leaf abscission-related genes and their functional characterization.

## Figures and Tables

**Table 1:** SLAF-seq data summary for the parents and F_1_ population of citrus.

Total Reads	489,334,306
No. of reads (♀)	7,664,165
No. of reads (♂)	8,095,110
No. of reads (F_1_, 1–200)	473,575,031
No. of high-quality SLAFs	257,409
Polymorphic SLAFs	84,525 (32.84%)
Non-polymorphic SLAFs	172,899 (67.16%)
Repetitive SLAFs	73 (0.03%)
Average depth in female parent	37.07×
Average depth in male parent	39.81×
Average depth in individuals	9.69×
No. of high-quality SLAF markers	4307

**Table 2:** Summary of the sex-specific linkage maps of citrus.

Linkage Group	Female-Specific Map				Male-Specific Map			
	Mapped Markers	Distinct Positions	Genetic Length (cM)	Average Interval (cM)	Mapped Markers	Distinct Positions	Genetic Length (cM)	Average Interval (cM)
1	106	54	123.48	2.29	376	125	162.89	1.02
2	168	61	160.78	2.64	447	164	181.71	1.11
3	373	69	98.64	1.43	707	218	233.97	1.07
4	193	34	108.42	3.19	311	107	110.31	1.03
5	90	61	89.41	1.46	71	30	44.10	1.47
6	359	102	155.96	1.53	324	111	114.30	1.03
7	94	33	132.05	4.00	374	116	141.15	1.22
8	36	27	120.76	4.47	39	27	85.10	3.15
9	59	36	104.40	2.90	327	119	153.50	1.29
Total	1478	477	1093.90	2.66	2976	1017	1227.03	1.38

**Table 3:** Summary of the integrated linkage map of citrus.

Linkage Group	Integrated Linkage Map						
	Mapped Markers	Distinct Positions	Genetic Length (cM)	Average Interval (cM)	Max Gap (cM)	Gap < 5 cM (%)	Segregation Distortion Ratio (0.001 < *p* < 0.05)
1	470	173	146.50	0.85	20.99	99.79	26.6
2	589	209	171.60	0.82	3.28	99.15	2.2
3	971	263	200.58	0.76	5.59	99.18	6.0
4	494	136	110.42	0.81	3.26	99.59	2.8
5	158	88	95.83	1.09	11.73	98.09	44.9
6	588	183	136.41	0.75	4.96	99.32	13.1
7	444	143	137.35	0.96	14.74	99.10	20.3
8	74	53	167.90	3.17	19.35	84.93	24.3
9	375	145	129.71	0.89	5.59	99.73	4.5
Average	-	-	-	1.12	9.94	97.65	16.1
Total	4163	1393	1296.3	-	-	-	-

## Supplementary Materials

The following are available online at https://www.mdpi.com/article/10.3390/ijms22115723/s1, Figure S1: SLAF number of eight distinct segregation types. The x-axis represents eight segregation types, the y-axis represents the number of SLAF markers. Figure S2: The distribution of SLAF and SSR markers in nine maternal linkage groups. Figure S3. The distribution of SLAF and SSR markers in nine paternal linkage groups. Table S1. Sequence information of the mapped SLAF markers. Table S2. List of segregation data of 4164 SLAF markers. Table S3. Identification of candidate genes from two QTL regions of the leaf abscission phenotype. Table S4. SNPs occurring in 5’-UTR, 3’-UTR and intron of candidate genes related to leaf abscission. Table S5. SNPs occurring in the exon of candidate genes related to leaf abscission. Table S6. InDel occurring in candidate genes related to leaf abscission. Table S7. Detection of candidate genes using the RNA-seq method. Table S8. Differently expressed candidate genes found using the by RNA-seq method. Table S9: The primers used in this study.
