# Supplementary material for: High-Density Genetic Map Construction and Identification of QTLs Controlling Leaf Abscission Trait in Poncirus trifoliata
Source: Int J Mol Sci. 2021 May 27;22(11):5723. doi: 10.3390/ijms22115723 (PMC8198561; doi:10.3390/ijms22115723)
Supplement: Supplementary file 1 [file ijms-22-05723-s001.zip › Supplementary Figures S1-S3.pdf]

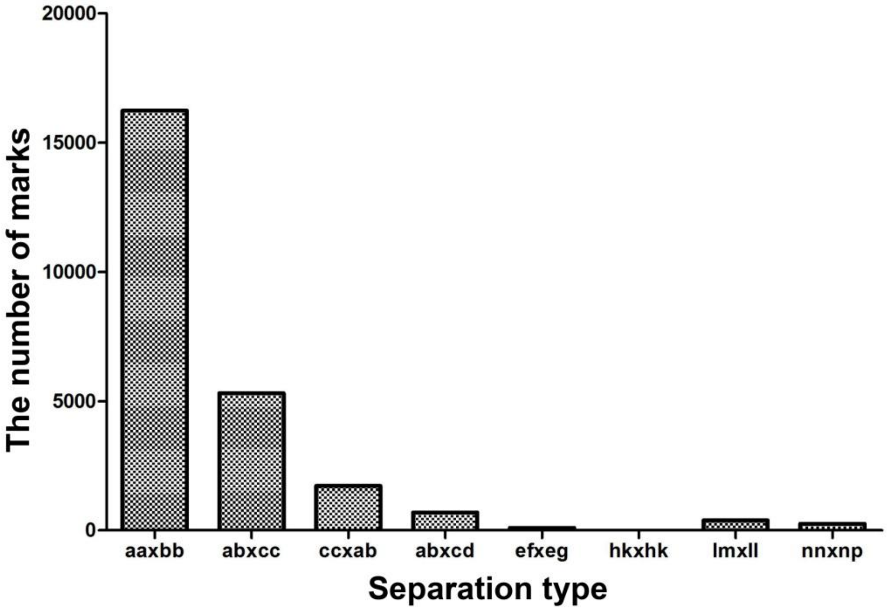


**Figure S1.** SLAF number of eight distinct segregation types. The x-axis represents eight segregation types, the y-axis represents the number of SLAF markers.

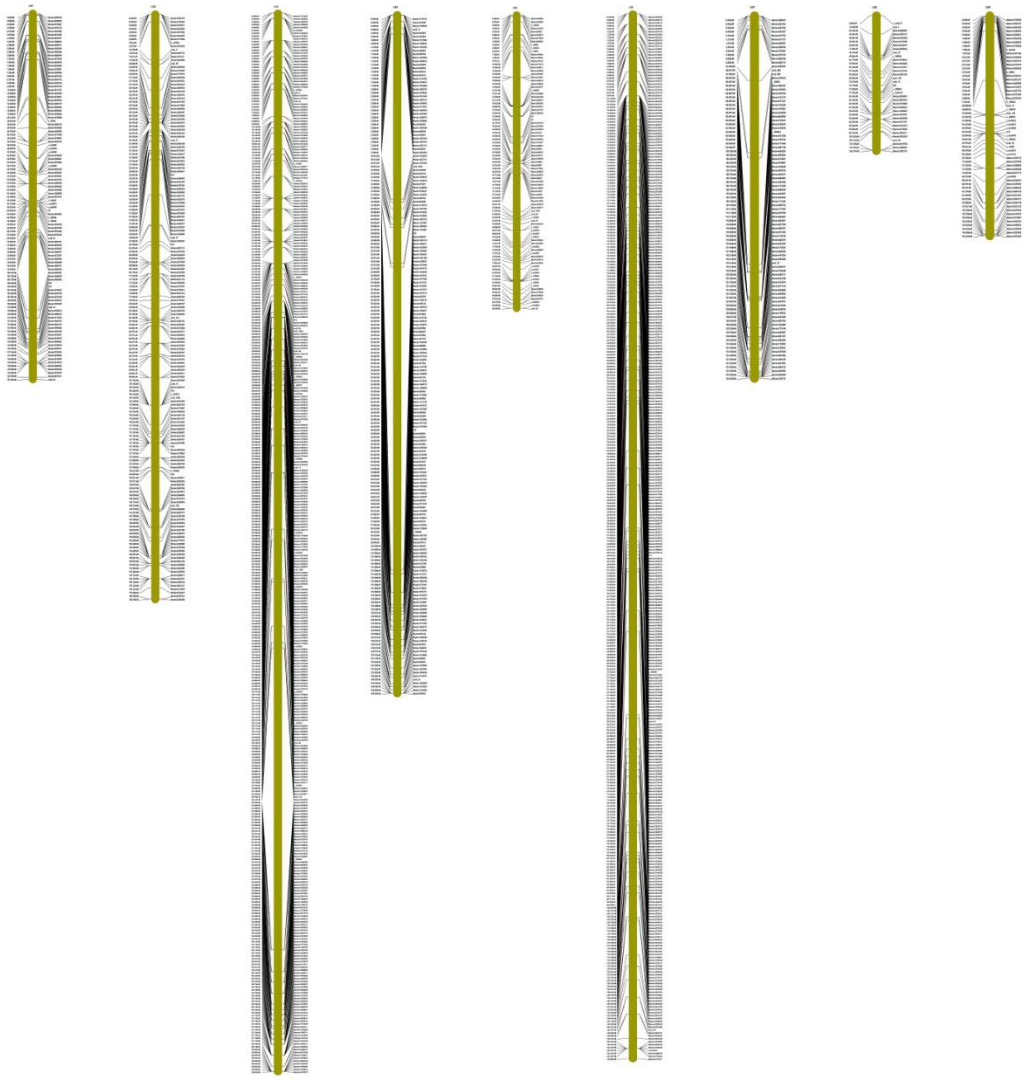

**Figure S2**. The distribution of SLAF and SSR markers in nine maternal linkage groups.

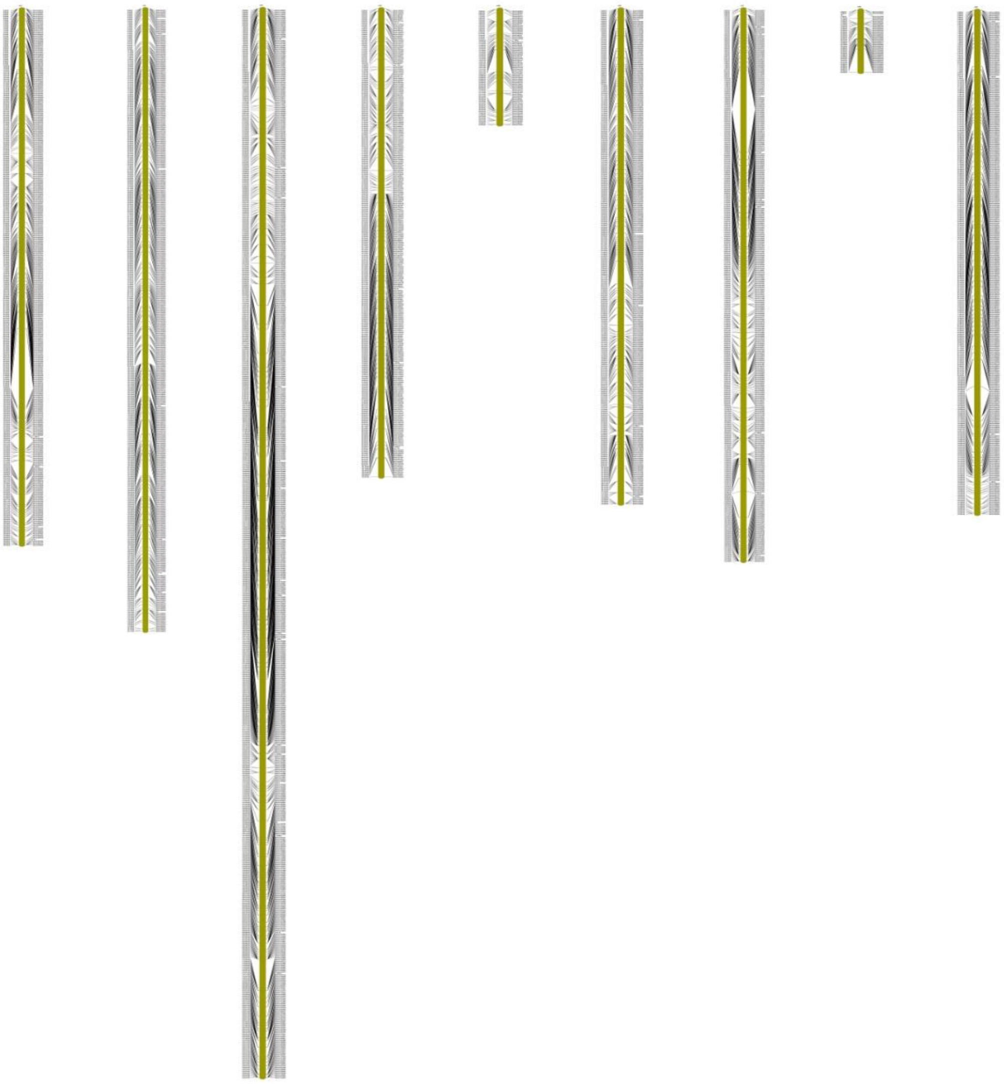

**Figure S3**. The distribution of SLAF and SSR markers in nine paternal linkage groups.
